# Automated deconvolution of structured mixtures from heterogeneous tumor genomic data

**DOI:** 10.1371/journal.pcbi.1005815

**Published:** 2017-10-23

**Authors:** Theodore Roman, Lu Xie, Russell Schwartz

**Affiliations:** 1 Computational Biology Department, School of Computer Science, Carnegie Mellon University, Pittsburgh, Pennsylvania, United States of America; 2 Biological Sciences Department, Mellon College of Science, Carnegie Mellon University, Pittsburgh, Pennsylvania, United States of America; Princeton University, UNITED STATES

## Abstract

With increasing appreciation for the extent and importance of intratumor heterogeneity, much attention in cancer research has focused on profiling heterogeneity on a single patient level. Although true single-cell genomic technologies are rapidly improving, they remain too noisy and costly at present for population-level studies. Bulk sequencing remains the standard for population-scale tumor genomics, creating a need for computational tools to separate contributions of multiple tumor clones and assorted stromal and infiltrating cell populations to pooled genomic data. All such methods are limited to coarse approximations of only a few cell subpopulations, however. In prior work, we demonstrated the feasibility of improving cell type deconvolution by taking advantage of substructure in genomic mixtures via a strategy called simplicial complex unmixing. We improve on past work by introducing enhancements to automate learning of substructured genomic mixtures, with specific emphasis on genome-wide copy number variation (CNV) data, as well as the ability to process quantitative RNA expression data, and heterogeneous combinations of RNA and CNV data. We introduce methods for dimensionality estimation to better decompose mixture model substructure; fuzzy clustering to better identify substructure in sparse, noisy data; and automated model inference methods for other key model parameters. We further demonstrate their effectiveness in identifying mixture substructure in true breast cancer CNV data from the Cancer Genome Atlas (TCGA). Source code is available at https://github.com/tedroman/WSCUnmix

## Author summary

One of the major challenges in making sense of cancer genomics is high heterogeneity cell-to-cell, as a tumor is typically made up of multiple cell populations with distinct genomes and gene expression patterns. The difficulty of working with such data has led to interest in computationally inferring the components of genomic mixtures. We develop a new approach to this problem designed to take better advantage of the fact that mixtures of cells across tumors or tumor regions can be expected to be highly non-uniform; samples that share greater common ancestry or progression mechanisms are likely to have more similar mixtures of cell types. We present new work on reconstructing mixtures from multiple genomic samples where the samples can be presumed to share such a pattern of similarity. Our methods automate the process of reconstructing these mixtures and the relationships between samples. We demonstrate their effectiveness on tumor genomic data in comparison to alternative methods in the literature.

## Introduction

Tumor heterogeneity is now recognized as a pervasive feature of cancer biology with implications for every step of cancer development, progression, metastasis, and mortality. Most solid tumors exhibit some form of hypermutability phenotype [1], leading to extensive genomic variability as tumor cell populations expand [2]. Studies of single cells by fluorescence in situ hybridization (FISH) [3, 4] have long revealed extensive cell-to-cell variability in single tumors, an observation that has since been shown, by single-cell sequencing technologies, to occur with a far greater scale and variety of mechanisms than previously suspected (e.g., [5, 6]). Furthermore, studies of clonal populations across progression stages have revealed that it is often rare cell populations that underlie progression, rather than the dominant clones [4]. Indeed, heterogeneity itself has been shown to be predictive of progression and patient outcomes [7]. All of these observations have suggested the importance of having ways of accurately profiling tumor heterogeneity, for both basic cancer research and translational applications.

Experimental technologies for profiling tumor heterogeneity are constantly improving, but are so far impractical for systematically profiling variability genome-wide in large patient populations. FISH and related imaging technologies can profile many thousands of cells, but only at limited sets of preselected markers [4]. Single-cell sequencing can derive genome-wide profiles of hundreds to thousands of cells in single tumors [5, 8, 9], but is so far cost-prohibitive for doing so in more than very small patient populations. Furthermore, technical challenges make it difficult to develop accurate profiles of structural variations, such as copy number variations (CNVs), which are the major drivers of progression in most solid tumors [10]. Bulk regional sequencing can profile small numbers of tumor sites per patient in large patient populations [11] but provides only a coarse picture of the heterogeneity within each site. RNA sequencing (RNA-Seq) provides a measure of the quantity of RNA expression and is practical on substantially larger numbers of single-cells than DNA-Seq [9]; however, it is subject to greater noise than DNA-Seq [12] and provides a more indirect measure of clonal heterogeneity.

These technical challenges to assessing heterogeneity experimentally have led to enormous interest in computational deconvolution (also known as mixed membership modeling or unmixing) methods as a way of computationally separating cell populations from mixed samples. Originally proposed as a way of correcting for stromal contamination in genomic measurements [13], such methods were later extended to reconstructing clonal substructure [14] and subclonal evolution [15] among tumor cell populations. The past few years have seen an explosion of such methods for deconvolution of numerous forms of genomic data sources (e.g., [16–31]). All such methods, however, are limited in accuracy and capable of resolving at best a few major clonal subpopulations, a small fraction of the heterogeneity revealed by single-cell experimental studies. These limits result from an inherent difficulty of separating high-dimensional mixtures, especially from sparse, noisy data. The gap between the heterogeneity we know to be present and what we can resolve by deconvolution is enormous, suggesting a need for further methodological advances.

Genomic deconvolution is a burgeoning field in which many different approaches are now available, often differing in models, algorithms, and the kinds of data or study design for which they are well suited. Leading contemporary approaches include TITAN [19], THetA [18], THetA2 [32], PhyloWGS [33], SPRUCE [34], Canopy [35], BitPhylogeny [36], and PyClone [37], each of which we briefly discuss here. TITAN uses a graphical model to estimate subpopulations based on copy number alterations and loss of heterozygosity events for whole genome or whole exome sequencing data, assuming as input read depths and allelic ratios at single nucleotide variant (SNV) sites. THetA and its follow-up version THetA2 perform tumor composition estimation using both SNV and copy number data derived from sequence read depths. PhyloWGS uses a probabilistic model to perform deconvolution jointly with phylogeny inference specifically on low-coverage whole genome sequencing data, making use of copy number estimates and variant allele frequencies (VAFs) of simple somatic variants. SPRUCE uses SNV and CNV data similar to that of THetA/THetA2 to make inferences as to the composition of heterogeneous tumor samples, but via a combinatorial enumeration strategy to explore the space of possible phylogenies consistent with a data set. Canopy optimizes for a probabilistic model to perform joint phylogenetic inference and tumor deconvolution from a data set based on several data sources, including VAFs and allele-specific copy numbers. BitPhylogeny similarly performs joint phylogenetics and deconvolution using Markov chain Monte Carlo (MCMC) sampling, but is unusual among methods in this domain in making use of DNA methylation data. PyClone performs tumor deconvolution for multiple samples from a single patient using SNV data, CNV data, and combinations thereof as input and is designed to work specifically with targeted deep sequencing data (>1000X coverage).

In prior work, we proposed that one could better resolve genomic mixtures by taking account of extensive substructure we would expect such mixtures to exhibit [21]. That is, an individual tumor or tumor site is not likely to be a uniform mixture of all cell types observed across all tumor samples in a study. Rather, one can expect distinct samples to group into subsets that share more or fewer cells depending on how closely related they are to one another. For example, all tumor samples can be expected to share some contamination by normal cells while tumors with common subtypes can be expected to share both normal cells and cell states characteristic of those subtypes. Likewise, tumor regions might be expected to share more similarity with those nearby than those more distant in a single patient. This kind of substructure is in principle exploitable to improve our ability to reconstruct accurate mixed membership models. Specifically, by deconstructing tumor samples into subgroups with similar mixtures, one can decompose the problem of reconstructing a high-dimensional mixture into the easier problem of reconstructing several overlapping lower-dimensional mixtures.

We previously showed how to implement such an approach to substructured mixture deconvolution, adapting an earlier deconvolution strategy for uniform mixtures that was based on identifying geometric structures (simplices) of tumor point clouds in genomic space [15, 38] but subdividing these point clouds into low-dimensional subsimplices that collectively constitute a higher-level object known as a simplicial complex. This prior work used a pipeline of several sequential steps to transform a genomic point cloud into a structured mixed membership model [21]:

1. Pre-processing / filtration
2. Dimensionality reduction
3. Pre-clustering (partitioning) into uniform submixtures
4. Unmixing submixtures
5. Unifying mixtures into a structured simplicial complex model

The resulting pipeline established a proof-of-concept for the approach, but also introduced several difficult computational challenges. For example, it required accurately pre-specifying the number of partitions and the dimensionality of each of the partitions, both difficult inference problems in themselves that require significant knowledge of the system under study.

In the present work, we improve on this proof-of-concept method by tackling several subproblems on the path to more completely automating inference of substructured genomic mixtures from populations of tumor samples. We have eliminated several nuisance parameters from the prior work, most notably by introducing methods for automated dimensionality estimation of subsimplicies and automated maximum likelihood inference of other previously user-defined parameters. We also improve upon our earlier work by proposing a model better suited to capture the uncertainty in cluster assignments through use of a fuzzy clustering representation of data points (samples) with respect to the inferred simplicial complex (and therefore the tumor phylogeny), allowing tumor samples to exhibit partial or uncertain membership in multiple phylogenetic branches. This flexibility is of particular importance when a sample is near a branch point in the simplicial structure, which corresponds biologically to a sample having a genomic profile similar to a most recent common ancestor of multiple tumor lineages. In addition, we develop a more comprehensive likelihood function, allowing us to optimize over and thus eliminate nuisance parameters from prior work.

Although the approach we introduce makes inferences as to intraturmor heterogeneity, we use information present across multiple patients (that is, intertumor heterogeneity) to make those inferences. This application assumes that commonalities in progression processes can be observed across subgroups of patients, even if the exact presentation is unique for each tumor. Because the model presumes common subgroups of tumors proceeding along similar evolutionary trajectories, an inferred mixture vertex will correspond to a coarse-grained model of a shared progression stage among a subset of tumors. That is, the vertex, would be interpreted as an approximate representation of a recurring cell type appearing in the course of progression of multiple samples. Since no two samples have exactly the same evolutionary history, however, it would be expected to reflect the common features of a cluster of similar cell types while averaging out their differences. The overall simplicial complex structure will correspond to a model of the space of evolutionary trajectories among all of these progressions stages across all observed tumor subgroups. Paths in the evolutionary tree will correspond to the recurring evolutionary pathways between the averaged progression stages represented by the vertices. Based on those reconstructions, we can then make inferences for each sample as to the relative amounts of each progression stage represented in that tumor, providing a coarse-grained inference of intratumor heterogeneity.

We validate the approach through application to breast tumor data from The Cancer Genome Atlas (TCGA) [39] and comparison with the widely-cited PyClone software [37]. We also compare with a more recent deconvolution method using DNA methylation data, providing an independent basis for comparison to the DNA copy number and RNA expression-derived deconvolution of our method [28].

## Materials and methods

**Fig 1:**
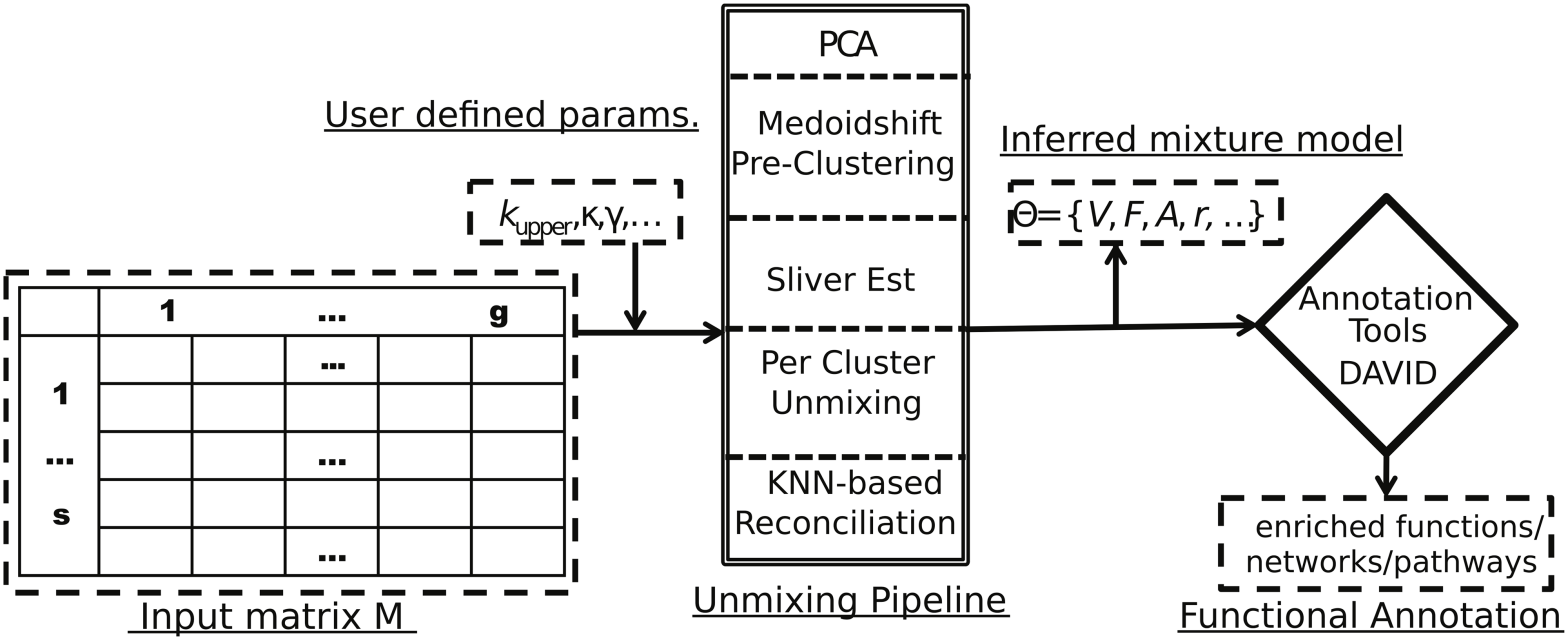
Overview of the full analysis pipeline: Input samples are represented by collections of copy number (CN) call files and/or RNA expression measurements, which are converted to a matrix format. These matrix inputs are passed to our simplicial complex inference code, which infers a mixed membership model of the data and associated model likelihood. The inference is computed by (1) principal components analysis (PCA) to perform dimensionality reduction and denoising of geometric structure; (2) medoidshift pre-clustering to identify low-dimensional sub-manifolds corresponding to distinct submixtures of the data; (3) dimensionality inference via sliver estimation to estimate likely numbers of mixture components needed to model each submixture; (4) unmixing on each substructure to identify preliminary mixture decompositions of the submixtures; and (5) a K-nearest-neighbor (KNN-based) reconciliation model to identify likely shared vertices between submanifolds. Each of these steps is explained in more detail in the main text. The inferred low dimension subspaces may be partially- or non-intersecting. We require, however, that the subspaces form a continuous structure, and merge disconnected subspaces using a maximum likelihood model. The inferred mixture components are then used in downstream functional annotation to identify dysregulated pathways or term associations.

In this section, we go through each step of our improved analysis pipeline, followed by a discussion of validation and application to real tumor data. We break the full inference problem into a series of sequential steps. Fig 1 provides a high-level overview of the process. The following subsections provide details on each component.

### Input and output data

We conceptually model input data as a matrix $M\in R^{s\times g}$, where the s∈N rows correspond to distinct samples (which might be biopsies of tumors in a patient population, tumor sites in a single patient, or regions of a single tumor) and the g∈N columns correspond to probes along a genome (typically one per gene, although potentially at lower or higher resolution). Note, however, that as the underlying data types input to the method are changed, the interpretation of output is changed correspondingly. For instance, if the features used as input are not gene copy numbers, but rather SNV sites, then the components of the matrix *M* will be SNV VAFs for the given samples. Similarly, if samples are different regions from a single patient, the inferred phylogeny is for a single patient, rather than across a patient panel. For ease of exposition, we refer to rows as samples and columns as genes below. We use this generic matrix format because data from many sources can be preprocessed into such a matrix (e.g., array-based CNV, SNV, or expression data or whole-genome or whole-exome sequence-derived CNVs, SNVs, or expression levels). Although the basic strategy is intended to be generic with respect to platform and genomic datatype, we specifically consider here three scenarios: 1) CNV data as might be derived from array comparative genomic hybridization (aCGH) or DNA-Seq read depths, 2) RNA expression data as might be derived from expression microarrays or RNA-Seq, and 3) a heterogeneous combination of DNA CNV and RNA expression data. Our goal is to decompose the rows of *M* into an approximately convex combination of a smaller set of unknown mixture components (putative cell populations). More formally, we seek a decomposition

M=FV+ϵ (1)

where $F\in R^{s\times k}$ are mixture proportions, $V\in R^{k\times g}$ are unmixed subpopulations, k∈N is the number of inferred cell subpopulations, and $\epsilon \in R^{s\times g}$ is an error matrix. *F* is interpreted as the mixture fractions of the pure subpopulations, also called mixing proportions, and *V* as the inferred genomic profiles of the pure subpopulations, also called mixture components. This interpretation leads to natural constraints on the problem: 1) ∑_*i*_ *F*_*ij*_ = 1 for a fixed *j* and 2) ∀*i*, *j*: 0 ≤ *F*_*i*, *j*_ ≤ 1. Given these constraints, the formal goal of the method is to compute *F* and *V* given *M*, with an intermediate step of determining the mixture dimension *k*.

Our approach to performing this deconvolution involves constructing a more involved simplicial complex mixed membership model, which will imply *F* and *V*, through a series of discrete inference steps. While most aspects of model inference are automated, as detailed in the remainder of Materials and Methods, the following parameters and hyperparameters still require manual selection:

1. Maximum number of dimensions for sliver estimation
2. Number of bootstrapped replicates for pre-clustering
3. Neighborhood size for pre-clustering
4. Number of nearest neighbors for vertex merger
5. Number of standard deviations for dimensionality estimation
6. Maximum number of iterations for fmincon

### Pre-processing

To begin analysis, we first pre-process *M* into a matrix of Z-scores:

$$M_{z}=\frac{M-\mu_{M}}{\sigma_{M}}$$ (2)

where *μ*_*M*_ is a vector of the mean copy numbers of each gene across all samples, and *σ*_*M*_ is a vector of the standard deviations of the copy numbers.

This process is altered slightly to accommodate heterogeneous DNA and RNA data that have been concatenated as features. We assume that the distributions of read counts will differ for DNA and RNA data, so instead of *μ* and *σ* for all samples column-wise, we use a *μ* and *σ* for pools of all data for each data type. That is, we evaluate the mean and standard deviation for Z-score computation for all samples and for all DNA features, and separately for all samples and all RNA features. In the RNA only case, we use the framework outlined in Eq 2. Next, to facilitate analysis of genomic point clouds, we reduce the dimension of the data using principal components analysis (PCA) [40]. While there are more sophisticated dimensionality reconstruction strategies available, we favor PCA as a simple, standard method that has relatively modest data needs. We identify a total of *k*_*upper*_ PCs, using the Matlab pca routine in economy mode, where $k_{upper}\in N<g$ is an upper bound on the number of cell subpopulations we will infer. In the present work, we use *k*_*upper*_ = 12, intended to be approximately an upper limit on the number of distinct mixture components a method of this class might be able to infer. We denote the PCA scores, corresponding to amounts of each PC in each tumor, as $S_{M}\in R^{s\times k_{upper}}$. Then, in order to fine-tune the automated dimensionality detection, we implement the sliver method of dimensionality estimation described in [41]. The core model proposed by that work relies on testing for the presence of “slivers”, geometric objects with poor aspect ratios, which occur when the following expression, which we call Assertion 3, is satisfied:

$$\begin{array}{r} \nu <\delta^{j}r\\ \text{where}\;r=\frac{L^{j}}{j!} \end{array}$$ (3)

where

- *ν* represents the volume of some enclosing structure,
- *j* represents the current estimate of dimension, increasing for each time Assertion 3 is false
  up until the limit of 12, and
- *δ* represents a tolerance factor between 0 and 1.

For a quick estimate of an enclosing structure, we use the algorithm proposed in [15]. We then use the top *j* − 1 PCs after the algorithm terminates. To automate the selection of the *δ* parameter, we use all values spaced 0.05 apart between 0 and 1. The range of possible *δ* values is 0 to 1 for this parameter based on the approach outlined by [41]. Because some values of the parameter lead to the same estimate of the dimensionality of the dataset, we choose one representative value from each partition of the range of dimension estimate values, then choose the model that has the highest likelihood.

Lastly, we normalize the scores for each PC to a [0, 1] range, which is then assumed by the pre-clustering technique applied in the next section [42]. We compute the 0–1 normalized version of *S*_*M*_ as

$$S_{\left[0,1\right]}=\frac{S_{M}-\text{min}S_{M}}{\text{max}S_{M}-\text{min}S_{M}}$$ (4)

where the minimums and maximums are computed for each PC, taken over all samples.

### Pre-clustering

We next pre-cluster data to identify initial candidate subsets of samples inferred to have drawn from the same set of mixture components. Each such subset will correspond to a distinct subsimplex of the full simplicial complex to be inferred. While this is a clustering problem, it is a non-standard one in that we seek to cluster data into distinct low-dimensional subspaces of a contiguous higher-dimensional point cloud, rather than into disjoint subclouds as is in conventional clustering. We developed a specialized clustering method for this purpose [42], based on a two-stage variant of medoidshift clustering [43]. We initially cluster in Euclidean PC space to reduce the raw data to a smaller set of representative data points. We then cluster these representatives under a negative-weight exponential kernel function using the ISOMAP distance measure [44], a form of geodesic metric measuring distance between data points through a k-nearest-neighbor graph of the input point cloud, which collectively draws on features of manifold learning and related technologies. The combination of ISOMAP distance and negative exponential kernel produces a clustering in which cluster representatives are approximately extremal points of the simplicial complex that serve to pull apart distinct subspaces of the point cloud. The initial Euclidean clustering suppresses noise, which otherwise makes the negative exponential kernel highly sensitive to outlier data. We refer the reader to [42] for full details. At the end of this process, we are left with a small set of cluster representatives *M*_2*stage*_, defined as the union over clusters *i* of a neighborhood *N*(*x*_*i*_) of points associated with each cluster representative *x*_*i*_:

$$M_{2stage}=\cup_{i}M_{N(x_{i})},$$ (5)

where each representative is itself a point in *S*_[0,1]_, and a corresponding clustering of all samples *C* = {*C*_1_, …, *C*_*r*_}, *S*_[0,1]_ = ⋃_*C*_*i*_∈*C*_*C*_*i*_.

We further assess uncertainty of the cluster assignments by determining a relative statistical weight of each data point in each cluster. We use a weight function based on a folded multivariate normal distribution, where the mean of the function is a 0 vector, the covariance matrix is the identity multiplied by the distance from each cluster center to the mean of all cluster centers, and the value at which the density function is evaluated is the distance from *x*_*i*_ to *C*_*j*_ in ISOMAP space. After these relative weights have been derived, we convert them to probabilities of assignment of each point to each cluster. If we denote the raw weight of the *i*^*th*^ data point as a vector *R*_*i*_, then we can define the normalized weight vector:

$$W_{i}=\frac{R_{i}-{\text{min}}_{C_{j}\in C}R_{i}}{{\text{max}}_{C_{j}\in C}R_{i}-{\text{min}}_{C_{j}\in C}R_{i}}$$ (6)

In the above formula, *C*_*j*_ refers to an arbitrary cluster in the clustering *C*, over which we maximize or minimize. The clustering in principle depends on a chosen neighborhood size for the k-nearest-neighbors graph, although a scan over all possible neighborhood sizes found no sensitivity of the final model likelihood to this parameter.

### Dimensionality inference in unmixing

We next seek to estimate the dimension of each cluster, which will correspond to the number of mixture components inferred for that cluster. The major challenge of this step is distinguishing a genuine axis of variation from random noise stemming from biological and technical limitations, particularly when working with sparse, noisy genomic measurements. Intuitively, we identify dimension by iteratively adding axes of variation via PCA until we can no longer reject the hypothesis that variance in the next dimension is distinguishable from noise.

We first build a model of expected noise per dimension by randomly sampling data points of pure Gaussian noise with mean 0 and identity covariance. We then perform PCA on this random point cloud and estimate the mean *μ*_*G*(*i*)_ and standard deviation *σ*_*G*(*i*)_ of the point cloud for each PC *i* ∈ 1, …, *k*_*upper*_. We then identify the smallest *i* ≤ *k*_*upper*_ such that the standard deviation of the true data in PC *i* is smaller than *μ*_*G*(*i*)_ + *κσ*_*G*(*i*)_, where *κ* defines a significance threshold in standard deviations. In the present work, we set *κ* = 3 to yield effectively a significance threshold of < 0.001 for rejecting the hypothesis that the next dimension can be explained by Gaussian noise. The result of this module, then, is a vector of inferred dimensions of each of the clusters: *D* ∈ {1, …, *k*_*upper*_}^*r*^. We would expect this test to be conservative (underestimate true dimension), although less so as the size of the data set and its precision increases. We found it necessary to use a custom-made conservative dimensionality estimator, as opposed to a more standard technique (e.g., [41]), because the number of data points available in this application is much smaller than is typically assumed by methods in this problem domain. We use the approach outlined in [41] in the initial phase, as it is prior to the pre-clustering, and therefore typically has a several-fold increase in the minimum number of data points considered, bringing it better in line with the data needs of that method.

### Cluster-wise unmixing

We next seek to establish an initial mixed membership model by separately unmixing each cluster, using the inferred dimension from the previous step as the number of mixture components. We establish the model by minimizing an objective function based on the noise-tolerant geometric unmixing method of [38]:

$$P(\theta |X)\propto \prod_{i=1}^{r}(exp(-\sum_{j=1}^{s}\left(|x_{i}-F_{j}^{i}V_{j}^{i}|W_{j}^{i}\right))MST{\left(V_{j},A_{j}\right)}^{-\gamma }\beta )$$ (7)

Where

- *γ* is a regularization penalty set based on an estimated signal-to-noise ratio (SNR) of the data source [21],
- *V* are the inferred vertices,
- *A* is the adjacency matrix,
- *MST* is a minimum spanning tree cost,
- *W* is the relative weight function computed above,
- *F* are the inferred mixture components,
- *x*_*i*_ is the *i*^*th*^ data point,
- *β* is a BIC penalty for model complexity [45] and,
- |⋅| is L1 distance.

The first term penalizes data points outside the bounding simplex via an exponentially-weighted L1 penalty. The MST term captures a form of minimum evolution model on the simplex itself intended to penalize the amount of mutation from a common source needed to explain the simplex vertices (mixture components) [21]. We optimize for the objective function via the Matlab fmincon function, fitting *V* and *F* to assign mixture components and mixture fractions to each cluster independently. In practice, we use a transformed version of the equation into negative log space, as the optimization packages are built for minimization rather than maximization, and log domain better handles underflow for small likelihoods while preserving the ordering of solutions.

### Reconciliation of subsimplices into a simplicial complex

We next seek to join the discrete simplices, each modeling a subset of samples as a uniform mixture, into a unified simplicial complex. We accomplish this by merging simplex vertices if we cannot reject the hypothesis that they represent distinct points in genomic space. We first establish a probability model using the k-nearest-neighbors graph on samples and vertices by modeling the set of overlapping neighbors between two vertices via a hypergeometric distribution. On the assumption two vertices draw their neighbor sets independently from the pool of all samples, the expected number of data points in common would be

$$\frac{\left|N_{1}||N_{2}\right|}{N}$$ (8)

where there are *N* data points, |*N*_1_| nearest neighbors of the first vertex, and |*N*_2_| neighbors of the second vertex. We merge two vertices when the number of observed overlapping nearest neighbors is above expectation. We empirically determined on our synthetic data that the method is insensitive to the number of nearest neighbors for choices between 2 and $\sqrt{N}$ and chose *k* = 15 nearest neighbors arbitrarily within this range for the real data. This approach replaces computationally costly bootstrap estimates used in our prior work [21].

**Fig 2:**
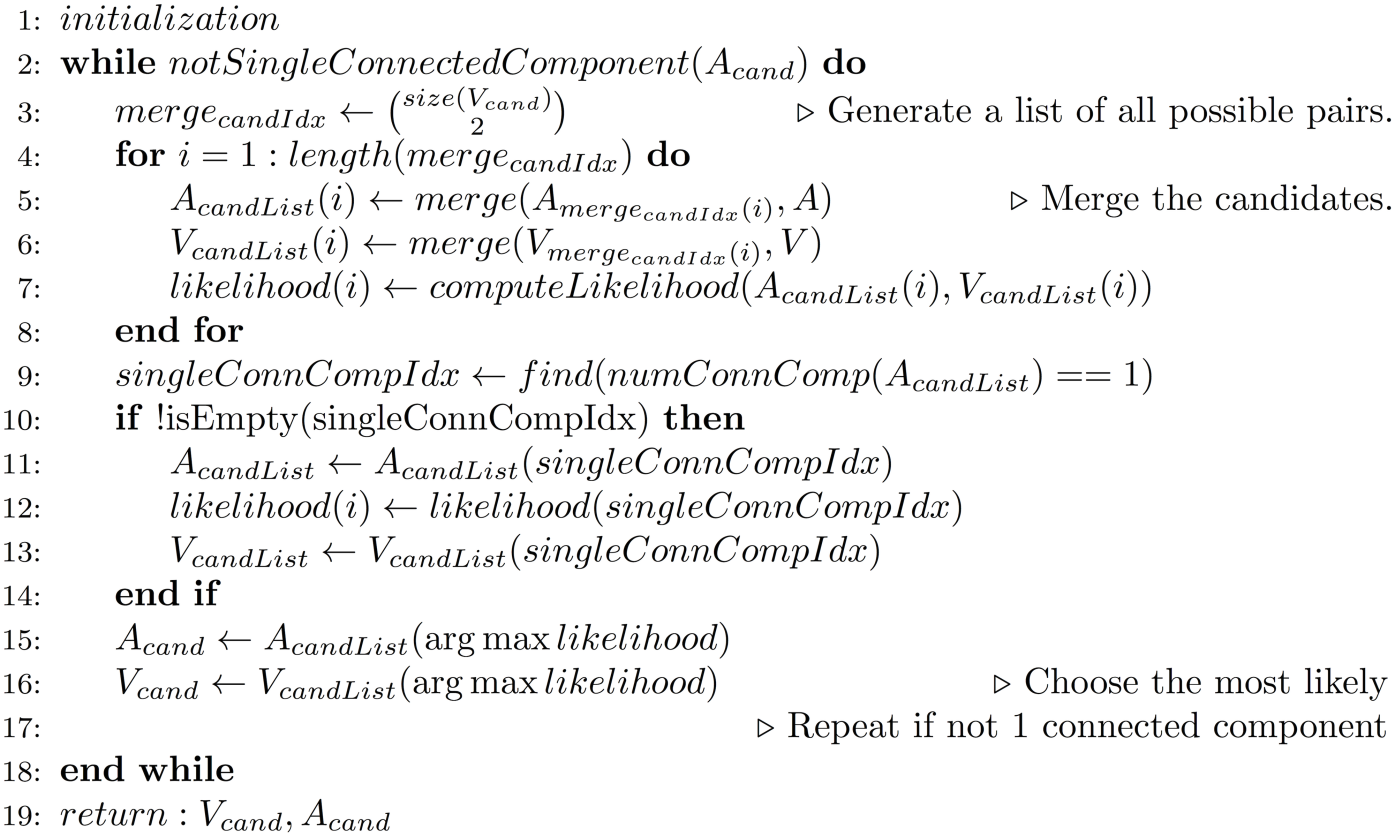
Pseudocode for merging protocol to select most likely from a set of candidate models provided none are simplicial complexes.

For those instances in which the process above does not result in a single connected simplicial complex, we add a step of post-processing to reconcile the geometric body into a single, connected simplicial complex. For those collections of bodies that do not consist of one connected component after the hypergeometric distribution correction, we iterate over all pairs of simplex vertices, merge the two vertices by creating a new vertex from the mean of the previous two vertices in all features, set the adjacency matrix to the union of the adjacency matrices of the two previous vertices, and compute the value of the objective function outlined in Cluster-wise Unmixing. We continue to merge points until there is at least one candidate consisting of a single connected component. If there are multiple such candidates, the candidate with the lowest objective function value, corresponding to the maximum of the likelihood function, is chosen. Pseudocode for this algorithm is provided in Fig 2.

### Application to TCGA tumor data

To demonstrate the efficacy of the algorithm, we use breast cancer (BRCA) CNV and RNA-Seq data from The Cancer Genome Atlas (TCGA) [39]. We downloaded level 4 DNA CNV data on 2 Jun 2016 (1,080 samples) and RNA-SeqV2 data on 1 Jun 2016 (1,041 samples), of which 1,022 samples were in common, along with clinical data for this cohort. For copy number data at level 4, gene features are extracted and a list of genes is provided, in contrast to the blocking procedure required by earlier work [42]; however, the platform is flexible to represent more or less granular data.

We ran the pipeline using the following parameters: maximum number of dimensions supplied to the pre-processing sliver method: 12; number of bootstrapped replicates for pre-clustering: 1000; neighborhood size for pre-clustering: 1; number of nearest neighbors for vertex merger: 15; cutoff for dimensionality estimation: 3 standard deviations; maximum number of iterations of fmincon per simplex: 1000. The choices reflect computational resource limitations, as well as a stable number of bootstrapped replicates, and choices to ensure convergence of the methods. The neighborhood size was chosen based on assumptions implicit in our normalization technique—for full details, see [42]. The number of nearest neighbors was chosen based on the test of simulated data similar to [42] demonstrating insensitivity to this parameter up to approximately $\sqrt{N}$ neighbors. The 3 standard deviations chosen correspond to a p-value of approximately 0.001. The runtime of the experiments depends largely on the dimension of the maximally likely clusters (i.e., the number of subpopulations in the tumor dataset that our model chooses as most likely) and the number of iterations in the minimization phase (iterations of fmincon).

### Sensitivity analysis

In order to assess the consistency of our method with respect to outlier data points, we conducted a sensitivity analysis using the TCGA CNV data. The sensitivity analysis was structured in an analogous fashion to 10-fold cross validation. For each of ten iterations, we excluded 10% of the data set, selected by a random uniform distribution. For the remaining data, the model was run to completion to produce a simplicial complex and assignment of mixture components and mixture fractions to the data points in that set of replicates. We then compared inferences by several measures to assess consistency across subsamples of the data.

We assessed similarity of the inferred component sets between replicates. To assess similarity of two sets of inferred vertex components A and B, we first identified for each component in A the closest matching component B, based on normalized Euclidean distance in PC space. We likewise identified for each component in B, the closest matching component in A. We assigned a score for the similarity of two vertex sets based on the mean distance between each component and its closest match relative to the mean distance between pairs of distinct components within A and within B.

## Results

To demonstrate the utility of our method, we consider three applications to data derived from the TCGA breast cancer cohort [46]. Breast tumors were chosen as an application case for two key reasons. First, there are a larger number of breast tumor samples than any other organ cancer type in TCGA, valuable for cross-cohort deconvolution. Second, breast tumors have well-documented clinical subtypes (HER2+; ER/PR+, and Triple Negative), a useful feature for validation since we would expect tumors and cell lineages within them to partition largely by subtype.

### Application to breast tumor RNA data

**Fig 3:**
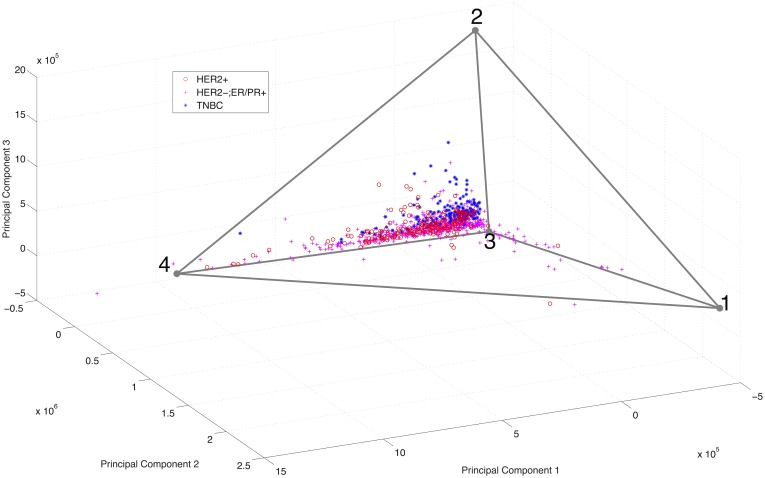
Visualization of TCGA RNA-Seq data with inferred maximum likelihood simplicial complex structure. Note that the tetrahedron inferred was considered alongside other simplices and simplicial complex but considered most likely. The data are enclosed in the tetrahedron, and as such can be approximated as mixtures of the vertices. Data points, corresponding to distinct tumor samples plotted in principal component space, are color coded by immunohistological subtype (red circle: Her2+, purple plus: ER/PR+, blue asterisk: triple-negative).

RNA-Seq data was downloaded from TCGA. The data consists of lists of gene expression in normalized counts, as well as gene name lists identifying each feature. Data from each of the samples were concatenated into a matrix of samples by genes. Using the parameters described above, the weighted unmixing procedure produces a tetrahedral simplex. Although other simplicies and simplicial complexes were considered by our algorithm, the tetrahedron was determined to be the maximum likelihood model. The results are illustrated in Fig 3, which shows the true point cloud as well as our inferred structure, where samples are colored by the clinical subtype. The DNA level 4 data consists of *log*2(⋅) copy number ratios, which are exponentiated and Z-scored prior to unmixing following the methods outlined above.

### Application to breast tumor DNA CNV data

**Fig 4:**
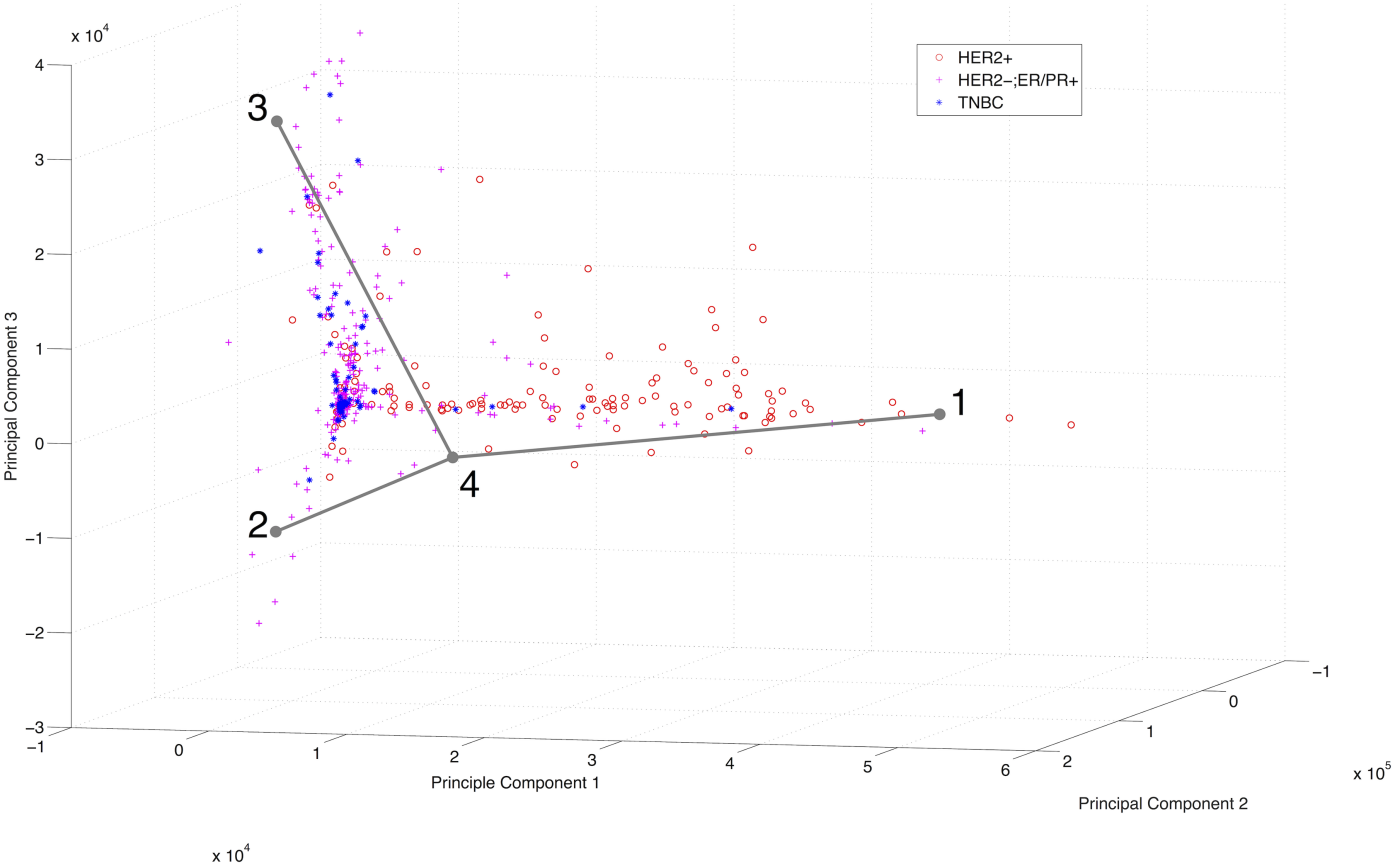
Visualization of TCGA CNV data with inferred maximum likelihood simplicial complex structure. The inferred structure of three arms sharing a point corresponds to a phylogeny of one most recent common ancestor, and three branches of a tree. Data points, corresponding to distinct tumor samples plotted in principal component space, are color coded by immunohistological subtype (red circle: Her2+, purple plus: ER/PR+, blue asterisk: triple-negative).

We also considered application to DNA CNV data from TCGA. The results are visualized in Fig 4. The decreased noise of DNA CNV technology relative to RNA-Seq technology results in a more sharply defined simplicial complex structure than was apparent with RNA-Seq data, consisting of three lines connected at a shared fulcrum. We attribute the clearer structure to the lower inherent stochasticity of DNA versus RNA data, which would be expected to better approximate the assumption that mixtures of cells will behave as linear combinations of their underlying cell types. We note that the central vertex, labeled 4, appears skewed away from the apparent junction of the three subsimplices. We attribute this skew in the position of the junction to the difficulty of accurately clustering samples near such subsimplicial boundaries, leading to imprecise positioning of the shared vertex in the distinct subsimplices that is only partly corrected when the vertices are merged.

### Application to breast tumor combined RNA and DNA CNV data

**Fig 5:**
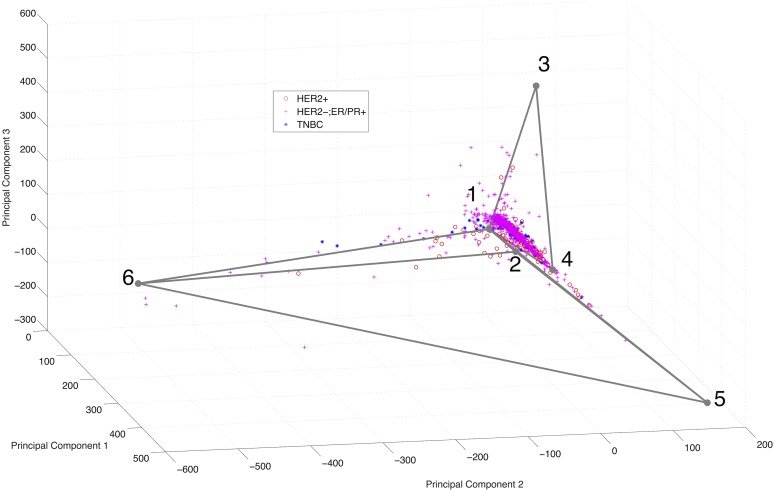
Visualization of TCGA combined RNA-Seq and CNV data with inferred maximum likelihood simplicial complex structure. The inferred structure of a tetrahedron and triangle sharing a point corresponds to two phylogenetic branches, one with four components and one with three components. Data points, corresponding to distinct tumor samples plotted in principal component space, are color coded by immunohistological subtype (red circle: Her2+, purple plus: ER/PR+, blue asterisk: triple-negative).

Lastly, we considered a combination of DNA and RNA features. Because of the varying noise profiles of the data types [12], we adjusted the normalization procedure as outlined above. We have plotted the results of the unmixing below in Fig 5, using the same color code for tumor subtypes as with the RNA-only and DNA-only data. The combined data leads to a somewhat more complex structure than either individual data type alone, consisting of a tetrahedron and triangle connected at a point. The higher dimension compared to the individual data types may reflect changes in the overall noise profile or to the complementary aspects of progression that are revealed by the two data types in isolation.

### Sensitivity analysis

We further used the TCGA CNV data to assess sensitivity of the method to subsamples of the data. We assessed reproducibility across ten replicates of 90% subsamples of the TCGA data and quantified reproducibility of inferred mixture component sets based on the ratio of Euclidean distances between best matching component pairs between replicates versus Euclidean distances within replicate sets. A score below one would then indicate general consistency between vertex sets relative to variability within each set, while a higher score would then be interpreted to mean that vertex components are highly distinct between runs relative to the variability among components within a set. Across all 45 comparisons among pairs of replicates, we found a mean distance of 0.6806 by this measure. This result suggests there is sensitivity to outliers in the simplicial inference leading to variability replicate-to-replicate, but that there is nonetheless similarity run-to-run relative to the variability in individual data sets.

### Ontological term enrichment

To assess the functional and biological significance of the inferences made by our model in each of the three test cases, we projected the data points from PC space back into genome Z-score space. We then identified genes lists with statistically-significant increase or decrease in Z-score as assessed by Bonferroni-corrected p-values. In the RNA and combined cases, we used *p* = 0.01 after correction. In the DNA case, at the *p* = 0.01 level, DAVID [47] reported that the number of genes provided was too large to process the results. As a result, we chose a stricter threshold of *p* = 2.1905 × 10^−11^, the smallest value we could choose without producing underflows in the p-value calculation.

Those genes that were statistically significantly upregulated were then evaluated on a per-vertex basis by DAVID [47] for enrichment by functional terms corresponding to specific networks, pathways, or other functional classes. In our case, we have specific interest in enriched tissues, diseases, and disease classes, as these areas provide the ability for the database to point specifically to our dataset. As expected, the DAVID analysis revealed enrichment for several terms related to breast cancer specifically, as well as breast tissue more broadly. In Tables 1–3, we provide the most significantly enriched terms for each of RNA, DNA, and combined RNA/DNA deconvolution. Tables 1 and 2 present the ten most significantly enriched terms for RNA and DNA, respectively. Complete lists of significantly enriched genes (p ≤ 0.05) appear in Supplementary Material as S1 and S2 Tables. Only seven terms were significantly enriched for combined RNA/DNA deconvolution and therefore only those are listed in Table 3. Comparison of the method shows that DNA-only results in the largest number of distinct pathways enriched, followed by RNA-only, then combined. Combined, however, is most specifically enriched for expected term classes broadly related to cancers and breast tissue. These results may suggest that the combined data is more effective at achieving high specificity at a trade-off in sensitivity.

**Table 1:** Top DAVID term enrichment results for RNA expression deconvolution. The table provides the ten most significantly enriched terms, identified by source repository and term, Benjamini-corrected p-values, and associated vertices of the inferred simplicial complex.

Source	Term	p-value	Vertex
GAD DISEASE CLASS	Immune	3E-010	3
KEGG PATHWAY	Allograft Rejection	4.7E-009	3
KEGG PATHWAY	Cell adhesion molecules	2.4E-009	3
KEGG PATHWAY	Graft-versus-host disease	3.8E-009	3
KEGG PATHWAY	Antigen Processing and Presentation	3.3E-009	3
Reactome pathway	Signaling in Immune System	2.8E-014	3
UP TISSUE	Spleen	1.9E-017	3
UP TISSUE	Blood	1.4E-012	3
UP TISSUE	B-Cell	1.1E-011	3
UP TISSUE	Lymph	3.1E-011	3

**Table 2:** Top DAVID term enrichment for DNA copy number deconvolution. The table provides the ten most significantly enriched terms, identified by source repository and term, Benjamini-corrected p-values, and associated vertices of the inferred simplicial complex.

Source	Term	p-value	Vertex
KEGG PATHWAY	Systemic lupus erythematosus	9.1E-012	1
KEGG PATHWAY	Alcoholism	1.4E-010	1
UP TISSUE	Blood	1.7E-005	1
UP TISSUE	Spinal cord	1.4E-005	1
UP TISSUE	Pancreas	0.0003	1
UNIGENE	Blood normal 3rd	0.00034	1
UNIGENE	mammary gland normal 3rd	0.00034	1
UNIGENE	ear normal 3rd	7.8E-005	3
CGAP SAGE	mammary gland breast carcinoma	4.4E-016	3
CGAP SAGE	liver poorly differentiated adenocarcinoma	8.8E-006	3

**Table 3:** DAVID term enrichment for combined RNA expression and DNA copy number deconvolution. The table provides significantly enriched terms, identified by source repository and term, Benjamini-corrected p-values, and associated vertices of the inferred simplicial complex.

Source	Term	p-value	Vertex
UNIGENE	Breast (mammary gland) cancer_disease_3rd	0.0083	2
GAD DISEASE CLASS	Cancer	0.0022	4
GAD DISEASE	breast cancer	5.1E-006	4
GAD DISEASE	Breast Cancer	1.1E-005	4
GAD DISEASE	Asthma—Autoimmune disease	0.0013	4
KEGG PATHWAY	Melanoma	0.036	4
UNIGENE EST	Breast (mammary gland) cancer_disease_3rd	0.02	4

### Comparison with prior methods

While there are many deconvolution tools in this domain, the variations in data assumptions of the methods make direct head-to-head comparison difficult. While TITAN [19] can make inferences from similar copy number variation data to our method, it depends on knowledge of alleleic frequency data at SNV sites unavailable to us in the present analysis. THetA [18] and THetA2 [32] perform a comparable form of inference but are tuned specifically for inference from a single tumor, making them unsuitable for comparison on a patient cohort for which our methods are designed. PhyloWGS [33] is designed for whole-genome analysis like our method, but depends on availability of variant allele fractions of novel somatic variants, a model and data type again unsuited to the kind of cross-cohort analysis performed by our method. SPRUCE [34] likewise depends on VAF data under the assumption that all samples are drawn from a single patient, making it poorly suited to the kind of data for which our method is designed. Canopy [35] likewise makes use of VAFs and allele-specific copy number data unavailable to us and poorly suited to the kind of cross-cohort analysis for which our method is designed. BitPhylogeny [36] likewise assumes a data type unavailable in our application, methylation data in that case, making direct comparison on real data infeasible.

In order to allow for some comparison to an alternative in the literature, we choose to compare to PyClone [37], as it is is a highly cited method producing similar output to our method that can in principle make inferences from a common set of data to our method. PyClone can work with copy number data and can optionally omit allele-specific frequency information (although it is designed to make use of such information if it is available). We emphasize that although PyClone can be run on a common data set to our method with some preprocessing, it is tuned for very different assumptions on those data than our method. PyClone assumes precise frequency estimates on small numbers of sites, as is appropriate to the targeted deep sequencing data for which it was designed, while our method is designed to use less precise data on large numbers of markers, as is appropriate for the whole exome or whole genome data for which it was designed. Furthermore, PyClone is also designed for multiple samples from a single tumor while ours is designed to work with cross-sectional data from distinct tumors. While we can run both methods on a common set of data, we thus cannot devise a single dataset that provides a fair test of both. Our intention in comparing the methods, then, is not to show that our method is superior to PyClone but rather that our method is filling a niche for which prior tools are not designed and to which they do not generalize well.

In order to preprocess the data in a format amenable to the PyClone system, we assume a read length of 300, and baseline copy number of 2. For this analysis, we assume a copy number of 2 for any region for which which there is no copy number alteration call in the data. We also omit analysis of sex chromosomes. We omitted allele-specific copy numbers as input to PyClone because this information is not part of the publicly-available version of TCGA data. Although both our approach and PyClone can run on SNV data, it proved computationally infeasible to include the SNVs in this dataset for the PyClone analysis, as PyClone is not designed to handle such a large marker set nor to work with markers drawn from many genetically distinct tumors.

We first attempted to run the full dataset of all level 4 gene copy number breast tumor samples from TCGA through the PyClone pipeline on a workstation equipped with an Intel i7-4770K processor at 3.5GHz per core, with 32GB of RAM. However, the approach was unable to complete in approximately 1 week of running time, which we inferred may be due to the large number of genes (> 20,000) present in the full dataset, an amount well in excess of the small targeted sequencing data assumed by PyClone, as well as by the fact the PyClone algorithm runs on a single core. We thus pruned the list of genes (features) to a subset of corresponding to known breast cancer driver genes from [48]. PyClone then successfully ran on the set of tumor data points from the TCGA breast tumor dataset. PyClone output also differs somewhat from that of our method, requiring some post-processing to facilitate comparison. PyClone outputs mean and variance scores for each sample, for each cluster of mutations, which can approximately relate to our vertices. Further, we consider a version of the means of the scores normalized to sum to one analogous to the mixture fraction scores we generate. We then test for similarities in Spearman correlation of our model’s inferred mixture fraction rank to the rank of mixture fraction provided by PyClone.

Results of comparison of our method with PyClone appear in Table 4. The PyClone comparison points (Py1 to Py4) correspond to the inferred mutational cluster prevalences. To make a fair comparison, we normalized the prevalences by their sums on a per-cluster basis to derive a fractional composition estimate based on PyClone. We then used Spearman correlation as a comparative tool to examine how similar in rank PyClone’s inferences of which clusters of genes were dysregulated are to our ranking of fractional composition with respect to inferred vertex amount. Because the vertices represent inferred pure subpopulations within tumor samples and are in PC space, the vertices are equivalent to genomic profiles of the subpopulations, where sets of genes are mutated. The correlation analysis provides a matrix of correlations where each element corresponds to the correlation between dysregulation of one Pyclone cluster and representation of one inferred subpopulation by our method. While in this case the two methods produced equal numbers of clusters, we would not expect that to be true for all data sets and the analysis does not assume the matrix dimensions are equal. There is significant (*p* < 0.01) positive correlation between 3 of our 4 vertices and 3 of the 4 clusters inferred by PyClone, signaling general agreement between methods in their estimate of substructure.

**Table 4:** Spearman correlation values (Rho values) among inferred vertices from simplicial complex unmixing by our method and subpopulation clusters derived from PyClone applied to TCGA breast cancer CNV data. The Py prefix is used for PyClone clusters. For our estimates, we use the V prefix. P-values for the comparisons appear in parentheses. Significant values (p<0.01) are marked in bold.

	Py1	Py2	Py3	Py4
V1	**-0.1269 (0.0012)**	**0.1305 (0.0009)**	**0.1137 (0.0037)**	0.0312 (0.4283)
V2	-0.0914 (0.199)	-0.0994 (0.0113)	-0.0869 (0.0269)	**0.209 (<0.00001)**
V3	-0.0265 (0.5002)	-0.026 (0.5083)	-0.0237 (0.547)	0.039 (0.3212)
V4	**0.204 (<0.0001)**	-0.0743 (0.0588)	-0.0383 (0.3298)	**-0.1501 (<0.00001)**

We further sought to validate our approach by comparing correlation of PyClone inferences to clinical labels supplied by TCGA (Table 5) with correlation of the inferences of our simplicial complex approach to the TCGA clinical labels (Table 6). Considering both positive and negative correlations, at the p≤0.0001 level, our approach has four entries significantly correlating to the clinical labels across two vertices, as compared to three entries across two clusters in the case of PyClone. Additionally, including those at a weakly significant (p ≤ 0.05) level, our approach has five entries correlating three of the four vertices’ mixture fractions to clinical subtypes, while PyClone has four entries across three of the four clusters’ mixture fractions to clinical subtypes.

**Table 5:** Spearman correlation from PyClone clusters derived from TCGA breast cancer CNV data to clinical labels of samples. P-values for the correlations appear in parentheses. Significant values (p<0.01) are marked in bold.

	HER2+	HER2-;ER/PR+	TNBC
Py1	-0.0756 (0.0543)	**0.1244 (0.0015)**	-0.0207 (0.5986)
Py2	**-0.1702 (<0.0001)**	0.0349 (0.3754)	0.0566 (0.1498)
Py3	-0.0383 (0.3307)	0.0709 (0.0711)	0.0343 (0.3835)
Py4	**0.2451 (<0.0001)**	**-0.2551 (<0.0001)**	0.0032 (0.9345)

**Table 6:** Spearman correlation of simplicial complex mixture fractions derived from TCGA breast CNV cancer data to clinical labels of samples. P-values for the comparisons appear in parentheses. Significant values (p<0.01) are marked in bold.

	HER2+	HER2-;ER/PR+	TNBC
V1	**0.1864 (<0.0001)**	**-0.1830 (<0.0001)**	-0.0533 (0.1752)
V2	0.0117 (0.7670)	0.0252 (0.5222)	-0.0026 (0.9467)
V3	-0.0782 (0.0467)	0.0215 (0.5853)	0.003 (0.9395)
V4	**-0.2027 (<0.0001)**	**0.1566 (0.0001)**	0.0711 (0.0706)

To provide an additional point of comparison, we also applied our method to TCGA RNA-Seq data. PyClone is not designed to accommodate RNA-Seq data, so we provide results only for our method. Table 7 shows the results. In this test case, the simplicial complex approach retrieved significant (p ≤ 0.01) correlation at each of the inferred vertices, and for each of the subtypes, with a total of 9 significant entries.

**Table 7:** Spearman correlation values for simplicial complex unmixing fractional estimates from TCGA breast cancer RNA-Seq data with TCGA-provided clinical subtypes. P-values for the comparisons appear in parentheses. Significant values (p<0.01) are marked in bold.

	HER2+	HER2-;ER/PR+	TNBC
V1	**-0.1304 (<0.0001)**	**0.2235 (<0.0001)**	**-0.2618 (<0.0001)**
V2	0.004 (0.8969)	**-0.2397 (<0.0001)**	**0.3131 (<0.0001)**
V3	**-0.1029 (0.0009)**	0.0033 (0.915)	0.0267 (0.3888)
V4	**0.1636 (<0.0001)**	**0.0979 (0.0016)**	**-0.1451 (<0.0001)**

While a perfect comparison of our method with PyClone, or any prevailing method known to us, is impossible given different data assumptions and input and output types, these comparisons provide clear evidence that our method is at least comparable in ability to identify substructure among tumor data sets when given appropriate input data to its model assumptions. On the whole, we interpret the results as being in agreement on the tested BRCA TCGA dataset, with our method providing the additional benefits of

1. Having the option to run on expression data, gene copy data, or heterogeneous combinations thereof,
2. Not requiring matched tumor-normal data or assumptions about normal samples, and
3. Being amenable to much larger numbers of features, such as might be derived from whole-genome data (WGS/WES), in comparison to PyClone or similar tools

In cases where the constraints of PyClone (deep targeted sequencing, matched tumor-normal samples) are well-satisfied, it may perform more accurately than the general approach we have developed, but in cases of lower read depth, datasets missing some or all normal matched samples, or with whole-genome coverage, the simplicial complex approach may be more appropriate.

We note that our method was not able to produce useful results on the trimmed list of genes we produced to yield a manageable gene set for PyClone. We speculate that the high noise in the data makes it infeasible to estimate simplicial structure from a small targeted gene set, resulting in our method fragmenting the samples into many more clusters. Our approach thus appears to be poorly suited to targeted gene sets and better to large or whole-genome data sets, in contrast to PyClone, which is well tuned for small numbers of genes but not computationally feasible for whole-genome data.

In order to further validate the approach, we examined Spearman correlation with an orthogonal data set. Onuchic et al. [28] developed a deconvolution approach based on DNA methylation data from TCGA [46]. The result of the Onuchic et al. [28] approach was a deconvolution of the data into constituent subtypes categorized into 5 cancer subgroups, a stromal group, an immune group, and a normal group. The results of correlating our results to theirs are shown in Table 8. There is significant (*p* < 0.01) positive correlation between what we estimate as the fulcrum of the simplicial complex—correspondening to the most recent ancestor in a phylogenetic interpretation—and the Onuchic et al. [28] estimate of stromal, immune, and normal composition. Further, our vertex 1 correlates in a statistically significant and positive way to their estimates of cancer subtype 1 and cancer subtype 5.

**Table 8:** Spearman correlation values for simplicial complex unmixing fractional estimates from TCGA breast cancer CNV data to fractional estimates, based on methylation data, from Onuchic et al [28]. P-values for the comparisons appear in parentheses. Significant values (p<0.01) are marked in bold.

	Cancer_1_	Cancer_2_	Cancer_3_	Cancer_4_
V1	**0.1026 (0.0009)**	-0.0151 (0.6261)	-0.0137 (0.6586)	0.0411 (0.1855)
V2	-0.0196 (0.5298)	0.03 (0.3341)	-0.0076 (0.8074)	0.0007 (0.982)
V3	0.047 (0.1306)	0.0133 (0.6697)	-0.0003 (0.9932)	0.0171 (0.5822)
V4	**-0.1562 (<0.0001)**	0.0018 (0.9545)	0.0118 (0.7041)	-0.0768 (0.0134)
	Cancer_5_	Stromal	Immune	Normal
V1	**0.091 (0.0034)**	-0.0666 (0.0321)	-0.0467 (0.1332)	-0.0399 (0.1989)
V2	0.0207 (0.5057)	-0.0489 (0.1156)	-0.0326 (0.2946)	-0.0199 (0.5224)
V3	0.0171 (0.5822)	-0.0726 (0.0194)	-0.0607 (0.0506)	-0.0433 (0.164)
V4	**-0.0855 (0.0059)**	**0.1197 (0.0001)**	**0.103 (0.0009)**	**0.1008 (0.0012)**

## Discussion

We have developed a novel method for taking better advantage of mixture substructure in deconvolution of mixed genomic data from heterogeneous tumor samples. This contribution is intended to advance a theoretical strategy for better resolving substructure in complex genomic mixtures, a general strategy that might be incorporated into many existing approaches for cell type deconvolution using assorted data types and inference models. The advances in the present paper bring us closer to the goal of deriving precise models of complex mixture substructure in the face of sparse, noisy genomic data without the need for extensive expert intervention. For this purpose, we have introduced new strategies for automated inference of subcluster dimensions, automated construction of a global simplicial complex structure, and better deconvolution of submixtures on small samples with uncertain subclustering. We have shown that we can automatically learn model structure from realistic sizes of data set without degrading performance of the model relative to methods requiring significantly more user intervention. We have further shown that this general approach is effective to varying degrees on CNV, RNA-Seq, and heterogeneous data sets. We have further shown that our method has comparable ability to resolve mixture structure to a leading deconvolution method, PyClone, on a common data set, while demonstrating several advantages in relaxing assumptions on data type, source, and quality.

The ultimate goal of the present work is to make sophisticated mixture deconvolution approaches more widely accessible to a non-expert community, by allowing them to be incorporated more broadly into a variety of deconvolution approaches in the literature. Much work still remains, though, both in better automating these approaches and improving inference quality. There are still several (hyper-)parameters for which the task of automated learning remains challenging. While automated dimension estimation appears valuable in improving simplicial complex models, deriving accurate estimates is a significant challenge for sparse, noisy data [49]. Integration of additional forms of genomic data into a common mixture framework is likewise a promising but challenging direction for improving inference quality. The computational framework presented here could also in principle be applied to many genomic samples from a single patient (e.g., distinct tumor regions, sites, or timepoints), although we do not explore that application here as data of this form is still scarce. The exact data needs of the method would depend on the heterogeneity across samples. We would expect this inter-sample heterogeneity to be substantially smaller for multiple samples from a single tumor than for the application to distinct tumors examined here, but nonetheless higher than is required for other tumor deconvolution methods that infer simpler underlying mixture models. Further, while we have applied this approach here to two data types and their combination, the same general strategy might be applied to many forms of genomic measurement (CNV, RNA expression, SNV, epigenetic, proteomic) and technologies for assessing them (array, sequence, or other high-throughput methods). Furthermore, as single-cell methods become more cost-effective, combinations of bulk and single-cell data may prove particularly informative. Finally, the simplicial complex models themselves require refinement to better capture the real sources of genomic mixture substructure they are meant to model, including substructure imposed by common pathways of subtype evolution, spatial constraints in the tumor microenvironment, and other sources of mixture substructure that do not conform well to our current simplicial complex model.

## Supporting information

S1 Table DAVID term enrichment for RNA expression deconvolution. The table provides significantly enriched terms (p ≤ 0.05), identified by source repository and term, Benjamini-corrected p-values, and associated vertices of the inferred simplicial complex.
(PDF)

S2 Table DAVID term enrichment for DNA copy number deconvolution. The table provides significantly enriched (p ≤ 0.05) terms, identified by source repository and term, Benjamini-corrected p-values, and associated vertices of the inferred simplicial complex.
(PDF)
