# Supplementary material for: Automated deconvolution of structured mixtures from heterogeneous tumor genomic data
Source: PLoS Comput Biol. 2017 Oct 23;13(10):e1005815. doi: 10.1371/journal.pcbi.1005815 (PMC5695636; doi:10.1371/journal.pcbi.1005815)
Supplement: S1 Table — The table provides significantly enriched terms (p ≤ 0.05), identified by source repository and term, Benjamini-corrected p-values, and associated vertices of the inferred simplicial complex. (PDF) [file pcbi.1005815.s001.pdf]

S1 Table. DAVID Term Enrichment for RNA Expression Deconvolution. The table provides significantly enriched terms ($p \leq 0.05$), identified by source repository and term, Benjamini-corrected p-values, and associated vertices of the inferred simplicial complex.

**Source**	**Term**	**p-value**	**Vertex**
GAD DISEASE CLASS	Immune	3E-010	3
GAD DISEASE CLASS	Infection	0.00052	3
BIOCARTA	Antigen Processing and Presentation	0.019	3
BIOCARTA	CTL mediated immune response against target cells	0.0057	3
KEGG PATHWAY	Allograft Rejection	4.7E-009	3
KEGG PATHWAY	Cell adhesion molecules	2.4E-009	3
KEGG PATHWAY	Graft-versus-host disease	3.8E-009	3
KEGG PATHWAY	Antigen Processing and Presentation	3.3E-009	3
KEGG PATHWAY	Type I diabetes mellitus	5.1E-009	3
KEGG PATHWAY	Viral myocarditis	4.7E-009	3
KEGG PATHWAY	Autoimmune thyroid disease	2.8E-008	3
KEGG PATHWAY	Intestinal immune netowkr for IgA production	2.9E-007	3
KEGG PATHWAY	Asthma	2.7E-005	3
KEGG PATHWAY	Systemic lupus erythematosus	0.00011	3
KEGG PATHWAY	cytokine-cytokine reception interaction	0.00084	3
KEGG PATHWAY	natural killer cell mediated cytotoxicity	0.00092	3
KEGG PATHWAY	hematopoietic cell lineage	0.0093	3
KEGG PATHWAY	chemokine signaling pathway	0.0094	3
Panther Pathway	T cell activation	0.00093	3
Reactome pathway	Signaling in Immune System	2.8E-014	3
UP TISSUE	Spleen	1.9E-017	3
UP TISSUE	Blood	1.4E-012	3
UP TISSUE	B-Cell	1.1E-011	3
UP TISSUE	Lymph	3.1E-011	3
UP TISSUE	Lymph Node	1.1E-005	3
UP TISSUE	Peripheral Blood	0.0001	3
UP TISSUE	T-Cell	0.00025	3
UP TISSUE	Leukocyte	0.00047	3
