# Supplementary material for: Automated deconvolution of structured mixtures from heterogeneous tumor genomic data
Source: PLoS Comput Biol. 2017 Oct 23;13(10):e1005815. doi: 10.1371/journal.pcbi.1005815 (PMC5695636; doi:10.1371/journal.pcbi.1005815)
Supplement: S2 Table — The table provides significantly enriched (p ≤ 0.05) terms, identified by source repository and term, Benjamini-corrected p-values, and associated vertices of the inferred simplicial complex. (PDF) [file pcbi.1005815.s002.pdf]

S2 Table. DAVID Term Enrichment for DNA Copy Number Deconvolution. The table provides significantly enriched ($p \leq 0.05$) terms, identified by source repository and term, Benjamini-corrected p-values, and associated vertices of the inferred simplicial complex.

Source	Term	p-value	Vertex
CGAP SAGE	stomach Adenocarcinoma 3rd	1.6e-3	1
CGAP SAGE	cartilage Dedifferentiated chondrosarcoma lung metastatis	5e-3	1
KEGG PATHWAY	Systemic lupus erythematosus	9.1E-012	1
KEGG PATHWAY	Alcoholism	1.4E-010	1
UP TISSUE	Blood	1.7E-005	1
UP TISSUE	Spinal cord	1.4E-005	1
UP TISSUE	Pancreas	0.0003	1
UP TISSUE	Ovary	0.0047	1
UP TISSUE	Mammary gland	0.004	1
UNIGENE	Breast (mammary gland) cancer disease 3rd	0.00057	1
UNIGENE	Blood normal 3rd	0.00034	1
UNIGENE	Ear normal 3rd	0.00043	1
UNIGENE	mammary gland normal 3rd	0.00034	1
UNIGENE	prostate normal 3rd	0.00043	1
UNIGENE	placenta normal 3rd	0.0013	1
UNIGENE	oral tumor disease	0.002	1
UNIGENE	spleen normal 3rd	0.0027	1
UNIGENE	respiratory tract tumor disease	0.0028	1
UNIGENE	bone normal 3rd	0.0032	1
UNIGENE	thymus normal 3rd	0.003	1
UNIGENE	uterus normal 3rd	0.0031	1
UNIGENE	colon normal	0.0043	1
UNIGENE	chondrosarcoma disease	0.0049	1
UNIGENE	adrenal gland normal	0.0056	1
UNIGENE	muscle tissue tumor	0.0073	1
UNIGENE	uterine tumor disese	0.005	1
UNIGENE	ovarian tumor 3rd	0.00046	2
UNIGENE	ovary normal	0.00046	2
CGAP Sage	mammary gland breast carcinoma	0.0048	2
CGAP SAGE	colon adenocarcinoma	0.0058	2
CGAP SAGE	mammary gland carcinoma metastasis to lung	0.0072	2
CGAP SAGE	liver poorly differentiated adenocarcinoma	0.0076	2
UNIGENE	ear normal 3rd	7.8E-005	3
UNIGENE	ovarian tumor 3rd	0.0012	3
UNIGENE	bone marrow normal	0.001	3
UNIGENE	tongue normal 3rd	0.0038	3
CGAP SAGE	mammary gland breast carcinoma	4.4E-016	3
CGAP SAGE	liver poorly differentiated adenocarcinoma	8.8E-006	3
CGAP SAGE	mammary gland carcinoma	0.0028	3
CGAP SAGE	prostate carcinoma	0.00037	3
CGAP SAGE	stomach, poorly differentiated carcinoma by surgery	0.0022	3
CGAP SAGE	thyroid follicular adenoma	0.0027	3
CGAP SAGE	liver	0.0032	3
